# Technical validation of a virtual reality-based eye tracker for neuro-ophthalmic assessment: a reliability and reproducibility study

**DOI:** 10.1038/s41598-025-30773-0

**Published:** 2026-01-05

**Authors:** Irem Karaer, Callum Hunt, Ha-Jun Yoon, Runfeng Ma, Reenette Savant, Vanessa Rodwell, Riddhi Shenoy, Zhanhan Tu, Qadeer Arshad, Elizabeta B. Mukaetova-Ladinska, Mervyn G. Thomas

**Affiliations:** 1 Ulverscroft Eye Unit, School of Psychology and Vision Sciences, University of Leicester, Leicester, LE2 7LX UK; 2 Chongqing Medical University Chongqing, Chongqing, China; 3 School of Psychology and Vision Sciences, University of Leicester, Leicester, UK; 4 The Evington Centre, Leicester General Hospital, Leicester, UK; 5 Department of Ophthalmology, University Hospitals of Leicester NHS Trust, LE1 5WW Leicester, UK

**Keywords:** Neuro-ophthalmic assessment, VR eye tracker, Feasibility, Reliability, Reproducibility, Motor control, Oculomotor system, Sensorimotor processing, Biomarkers, Health care

## Abstract

Virtual Reality (VR) eye trackers provide portable and objective tools for neuro-ophthalmic testing. This study aimed to assess the reliability and reproducibility of an emerging VR-based eye tracker (BulbiCAM) in healthy participants and compare its utility to an existing wearable eye-tracking system (PupilLabs Neon), thereby laying the groundwork for the future studies of clinical feasibility. Thirty-nine healthy participants underwent BulbiCAM testing across two visits, with inter-visit reproducibility evaluated using intra-class correlation coefficients. Pupillary light reflex assessments were conducted with both devices, allowing paired comparisons. Participant feedback demonstrated high acceptability, with 89% reporting the test as comfortable and 81.5% experiencing no eye strain or fatigue. BulbiCAM showed high reproducibility for pupil and pursuit tests (ICC = 0.76–0.88), though saccade reproducibility was lower (ICC = 0.46–0.62) which indicates limited reliability for saccade metrics. Key pupillometric parameters showed strong agreement between devices, with minimal bias observed in baseline diameter (-0.48 mm), peak constriction (-0.56 mm), constriction velocity (0.22 mm/s), and duration (-0.052 s). These findings support the potential clinical feasibility and reliability of BulbiCAM for both research and patient testing, offering a promising alternative for objective neuro-ophthalmic assessment.

**Supplementary Information:**

The online version contains supplementary material available at 10.1038/s41598-025-30773-0.

## Introduction

Eye-tracking devices are widely utilized in both neuro-ophthalmic research and clinical settings, providing valuable insights into ocular and neurological function^1–3^. In recent years, Virtual Reality (VR) headset eye-tracking devices have gained increasing popularity as portable and efficient alternatives to conventional eye-tracking systems^4^. By integrating advanced eye-tracking capabilities within VR headsets, these devices offer enhanced flexibility and convenience^5^. Unlike conventional setups with separate displays, projectors, and computers, these all-in-one systems offer a space-saving solution for clinics while providing accurate eye-tracking data.

Both subjective and objective methods are employed to evaluate eye movements and pupillary light reflexes. However, there is a growing need for clinically adaptable eye trackers utilizing objective methods^6^. Particularly, VR headset eye-tracking devices equipped with automated software, offer significant advantages by providing precise and standardized measurements, ensuring accuracy and reliability^7,8^. BulbiCAM is an example of a VR headset with eye tracking capabilities aimed at obtaining objective metrics for oculomotor behaviour and pupillometer; thus, having the potential for assessing a range of neuro-ophthalmic diseases and dysfunctions^9^.

Whilst previous research has examined VR headsets for eye tracking and pupil metrics^10,11^, our study places a specific emphasis on the BulbiCAM device, investigating not only the inter-test reproducibility of its saccade, pursuit, and pupillometry measures, but also its reliability compared to other wearable eye trackers with pupillometry capabilities (PupilLabs Neon glasses). Moreover, we addressed participant experience to engage with the BulbiCAM system’s comfort and acceptability, which is rarely reported. By evaluating these measures within a healthy population, this study lays important groundwork for future studies of BulbiCAM’s clinical adaptability.

## Materials (Subjects) and methods

### Participants

Healthy participants (*n* = 39) were recruited for the study and successfully completed both visits. Healthy subjects were defined as participants who had no neurological diseases or clinical signs of ocular motility dysfunction. By restricting inclusion criteria to healthy participants, assessment of technical validation and potential clinical application were aimed. The demographic characteristics of the participants are presented in Table 1. Their ages ranged from 21 to 59 years, and the male-to-female ratio was 18:21. Refractive errors ranged from − 8.00D to + 5.50D. All subjects achieved a best-corrected visual acuity (BCVA) of 0.20 logMAR or better. Colour vision, assessed with the Ishihara pseudoisochromatic plates, was normal (17/17) in most participants. However, one individual recorded scores of 5/17 in the right eye and 6/17 in the left eye.

None of the participants had a history of neurological disorders. Ophthalmological histories were largely unremarkable, except in five individuals: three had corneal refractive surgery, one had previously undergone strabismus surgery and had colour blindness, and one had lattice degeneration and posterior vitreous detachment in both eyes.

Written informed consent was obtained from each participant.

**Table 1:** Demographic information of participants.

Variable	
Age, Mean ± SD	30.2 ± 9.7
Sex, No.(%)	
**Female**	**21(54)**
**Male**	**18(46)**
**Ethnicity**,** No. (%)**	
**White-British**	**2 (5)**
**White-Other**	**8 (18)**
**Asian-Oriental**	**21 (54)**
**Asian-Indian Subcontinent**	**7 (18)**
**Afro-Caribbean**	**1 (2.5)**
**Other**	**1(2.5)**
**Visual Acuity (corrected**,** logMAR)**,** Mean ± SD**	
**Left eye**	**−0.01 ± 0.06**
**Right eye**	**−0.02 ± 0.07**
**Colour Vision**	
**Normal No. (%)**	**38 (97.4)**

### Study design

To assess the reproducibility of BulbiCAM measures and to enable comparison with a second device (PupilLabs Neon), a total of 39 participants were recruited for two study visits. Participants were scheduled at similar times of the day, but with no fixed interval between visits due to participant availability. Within this group of 39 participants, the median time between visits was 7 days (IQR = 11.5). At baseline, all participants underwent best-corrected visual acuity assessments (ETDRS chart at 4 m) and Ishihara tests for colour vision screening.

**Fig. 1:**
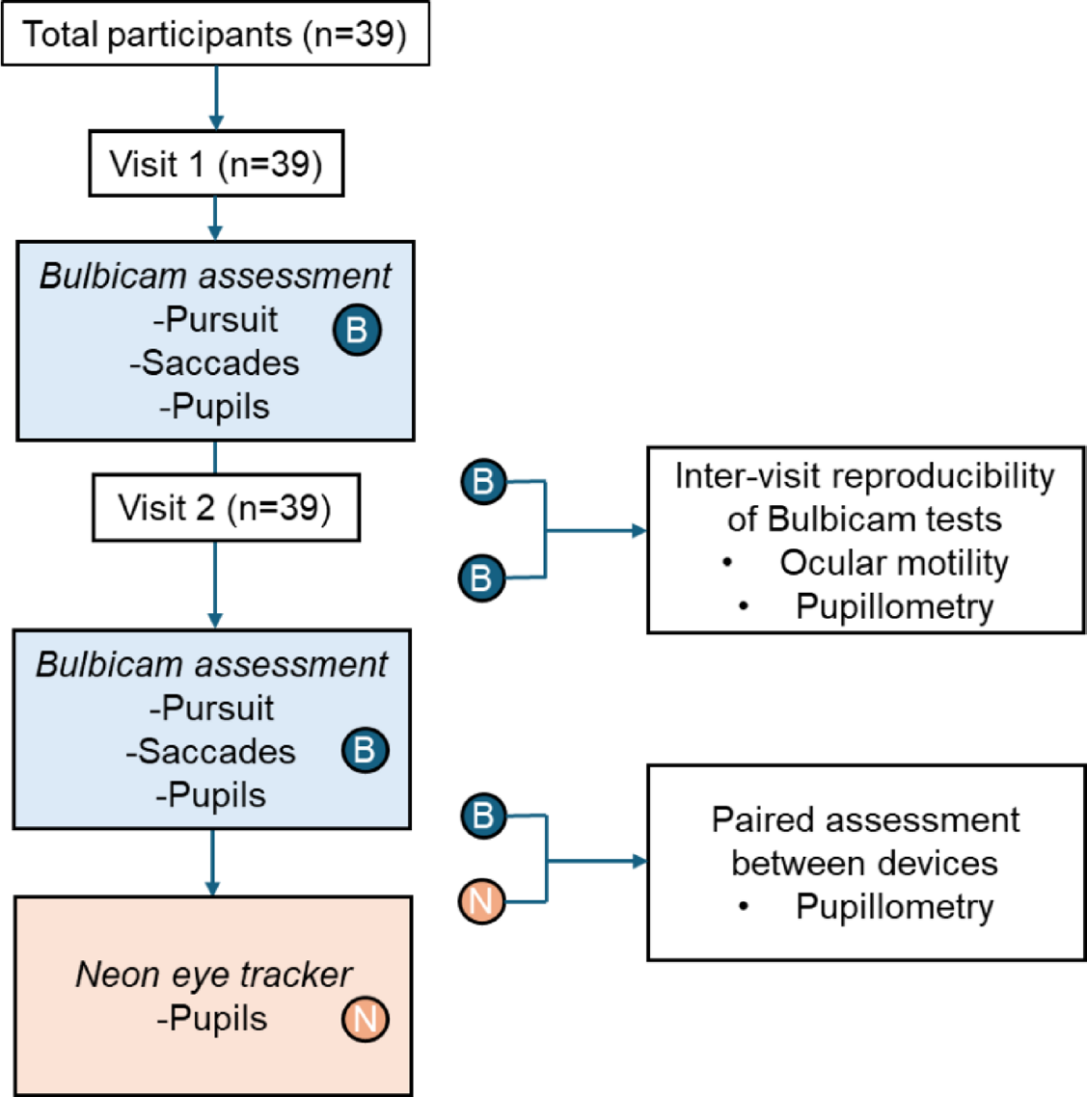
Flowchart explaining the study design. B: Bulbicam, N: PupilLabs Neon eye tracking glasses.

Figure 1 illustrates the participant flow through the study and the order of testing performed. Further details regarding the stimulus parameters used in BulbiCAM are provided below.

In the portion of the study involving Neon glasses, participants wore the glasses while tracking stimuli displayed on the BulbiCAM screen. For these measurements, participants’ heads were positioned slightly behind the standard posture to accommodate the glasses and ensure accurate data capture.

### Participant experience

Participants were asked to complete a questionnaire after completing two visits. The questionnaire scored^1^ (1) Comfort,^2^, (2) Length of the examination, ^3^, (3) Brightness,^4^, (4) Double vision^5^, (5) Grading the fatigue/eye strain specifically for Bulbicam on a 5-point Likert scale as: 1 = Strongly Disagree, 2 = Disagree, 3 = Neutral, 4 = Agree, 5 = Strongly Agree (Fig 3). 

**Fig. 2:**
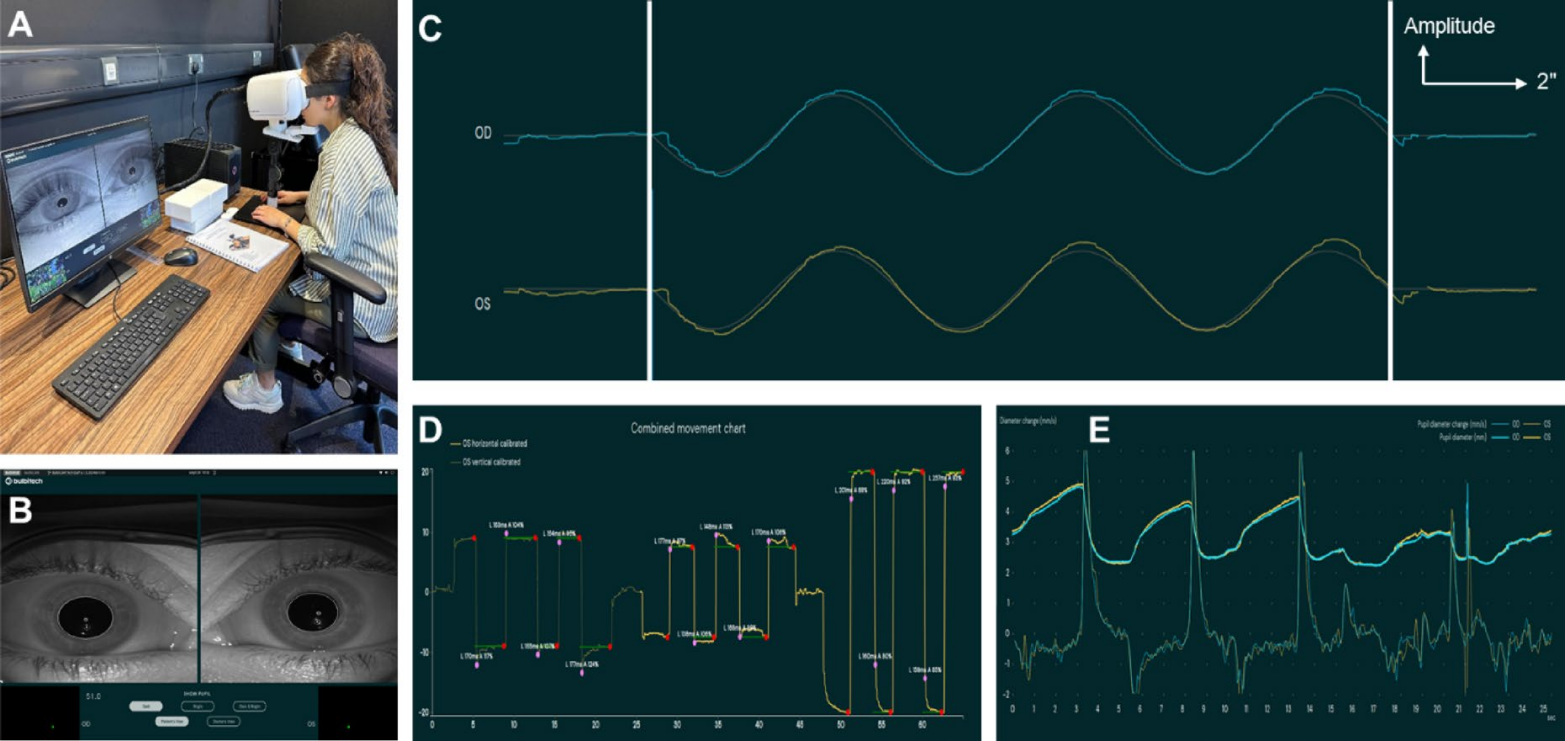
BulbiCAM Set up and Representative Test Outputs. (**A**) BulbiCAM eye tracking device set up and a participant being assessed (informed consent was obtained to publish the image in an online open access publication) (**B**) BulbiCAM screen during assessment (**C**) original pursuit test data. White lines show the starting and ending of the stimulus. Vertical arrow is a scale of wave amplitude and horizontal arrow represents two seconds (**D**) original saccades test data (**E**) original pupil test data.

### BulbiCAM

BulbiCAM (BulbiTech AS, Oslo, Norway) is a CE-certified and FDA-registered eye-tracking device that uses AI-integrated software to assess the data obtained by cameras. It accommodates an interpupillary distance of 49 mm to 75 mm. The display mode hardware utilised is the Sharp LS055R1SX04, which consists of 5.5” LCD panel (2560 × 1440 pixels @60 fps; 1920 × 1080 pixels @60 fps), MIPI board, White 60-pin flex cable, HDMI-MIPI board (based on TC358870XBG M silicon). The camera is configured to capture two different types of images alternately, Dark Pupil (DP) and Bright Pupil (BP). If the initial frame is BP, the next one will be DP, followed by BP again, and so on. The camera operates at 400 frames per second (FPS), capturing 200 DP and 200 BP images per second, resulting in a total of 400 pupil images per second. The stimulus synchronization typically has an accuracy of ± 18 ms, with a worst-case deviation of ± 38 ms^9^. Experimental set-up for BulbiCAM is shown in Fig. 2.

Detailed information on technical validation metrics, including detailed analyses of spatial accuracy and precision, temporal resolution, calibration accuracy, and AI-based pupil detection performance (sensitivity, specificity, and failure mode analysis), are provided in the Supplementary Information.

**Fig. 3:**
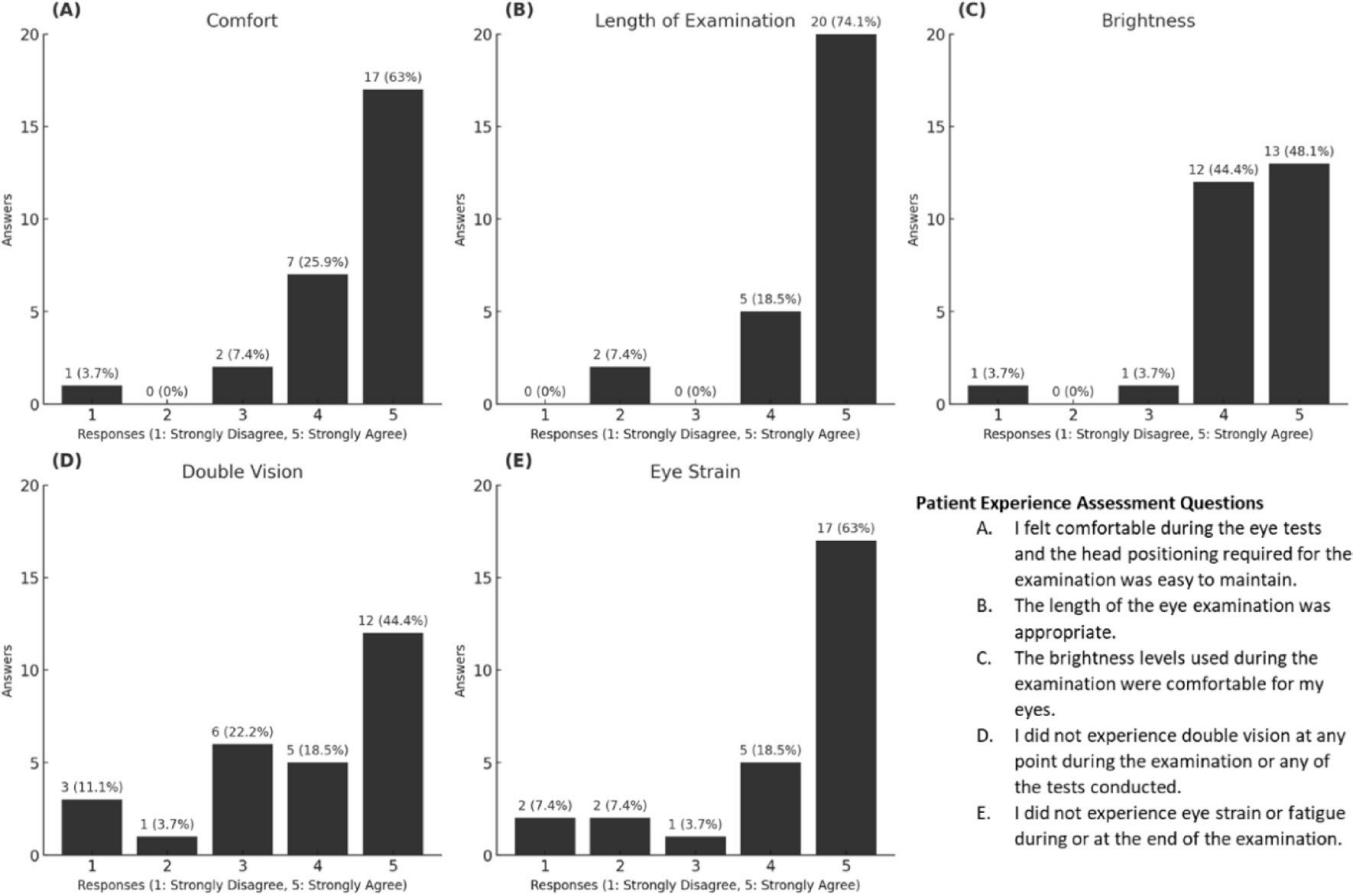
Patient Experience Across Different Variables. Bar charts illustrating the frequency of responses for various patient experience factors: (**A**) Comfort during the procedure, (**B**) Appropriateness of the examination length, (**C**) Brightness levels used during the examination, (**D**) Incidence of double vision, and (**E**) Eye strain or fatigue experienced during or after the examination.

### PupilLabs neon glasses

PupilLabs Neon Glasses (Pupil Labs GmbH, Berlin, Germany) are eye-tracking glasses equipped with scene cameras in front of the glasses and two eye cameras with 850 nm infrared illuminator LEDs. The device is connected to the companion device (Android phone) which enables the glasses to run and process the data collection. Pupil Cloud website is the recommended tool for analysing the data and processing the recordings. PupilLabs Neon glasses enable researchers to collect gaze, saccades, pupil diameters data alongside real-world front camera footage. The camera operates at a frequency of 200 FPS^12^.

### Data set

Pursuit, saccade, and pupillary light reflex values were obtained using BulbiCAM.

For pursuit, smooth pursuit testing was performed to assess the ability of the eyes to track a moving target smoothly. Gain values were recorded in response to 10° stimuli at 0.5 Hz. Gain, defined as the ratio of stimulus velocity to eye velocity, reflects tracking accuracy (100% indicates perfect tracking). By examining gain across different stimulus frequencies, the accuracy and robustness of the pursuit system can be evaluated under varying conditions^9,13^. Representative pursuit stimuli and recordings from the right and left eyes are shown in Fig. 2C.

For saccades, testing evaluated rapid eye movements that redirect gaze between points of interest and serve to quickly reorient the gaze toward objects during tasks such as visual search. Horizontal and vertical stimuli were presented, and participants were instructed to fixate on the target upon its appearance. Parameters measured included peak velocity, latency, and accuracy. Peak velocity represents the maximum eye speed during the saccade, latency reflects the delay between stimulus onset and saccade initiation, and accuracy indicates how closely the eyes landed on the target. These metrics provide quantitative information for both research and clinical assessment, particularly in identifying abnormal saccadic patterns such as hypermetria or hypometria. In accordance with BulbiCAM’s standard protocol, five trials of 9° vertical saccade (ranging from + 9° to −9°, indicating upward and downward directions respectively) task of and five trials of 20° horizontal saccade (ranging from − 20° to + 20°, indicating leftward and rightward directions respectively) task were assessed. Examples of horizontal and vertical saccades are shown in Fig. 2D.

Pupillometry testing evaluated the pupillary light reflex. Baseline and peak constriction diameters, as well as constriction velocity, were recorded. Each eye was exposed to varying screen brightness levels (3.3 cd), and changes in pupil diameter were measured throughout the trials. Because participants were healthy and a strong correlation between eyes was expected, only one eye was analysed. Figure 2E illustrates the dynamic pupil responses, highlighting constriction and subsequent dilation phases following light stimulation.

Alongside the BulbiCAM device, the same pupillometry variables were also collected using the Pupil Labs Neon glasses via the Pupil Cloud platform, enabling comparison of outputs between the two eye-tracking systems.

### Statistical analysis

A sample size calculation was performed based on an expected intraclass correlation coefficient (ICC) of 0.675, derived from saccadic gain reliability (ICCs ranged from 0.52 to 0.83) data in healthy controls reported in a Huntington’s disease study^14^. Assuming a null hypothesis ICC of 0.50, a significance level of 0.05, and 80% power, the required sample size was estimated to be 27 participants. To account for an anticipated 10% dropout rate, the final adjusted sample size was determined to be 31 participants.

The ICC was used to assess inter-visit reproducibility of BulbiCAM tests^15^. At the end of the two visits, data from *n* = 39 participants was analysed for the pursuit test, *n* = 37 for the vertical 9 degree saccadic test, *n* = 35 for the horizontal 20 degree saccadic test and *n* = 35 for the pupil test, respectively. Test reproducibility is categorized as excellent when the ICC exceeds 0.90, good when it falls between 0.75 and 0.90, moderate for values ranging from 0.50 to 0.75, and poor when it is below 0.50^16^.

We obtained paired pupillometry data from PupilLabs Neon glasses in *n* = 23 participants and used Bland-Altman test to evaluate the agreement in pupillary light reflex measurements between BulbiCAM and PupilLabs Neon glasses^17^.

Bland-Altman analysis was performed using GraphPad Prism 10.2.3, and ICC analysis using R 4.4.1.

### Ethical approval

This study received ethical approval from the East Midlands – Nottingham 2 Research Ethics Committee (REC) (REC reference: 21/EM/0017, protocol version: 0772, and the UK Health Research Authority (HRA) approval (IRAS ID: 279309). All research procedures were performed in accordance with relevant guidelines and regulations, including the Declaration of Helsinki. Informed consent was obtained from all participants and/or their legal guardian(s) prior to their inclusion in the study. Where applicable, additional consent was also obtained for the publication of identifying images in an online open-access journal.

## Results

### Participant experience

A total of *n* = 27 participants provided feedback on their BulbiCAM examination experience using a 5-point Likert scale, achieving an excellent response rate, with majority reporting positive experiences. Specifically, 89.0% found the test comfortable, 92.6% considered the examination length appropriate, and 92.5% were satisfied with the brightness levels. Furthermore, 62.9% reported no occurrence of double vision, and 81.5% indicated they experienced no eye strain or fatigue (Fig. 3).

### Inter-visit reproducibility of bulbicam tests

**Fig. 4:**
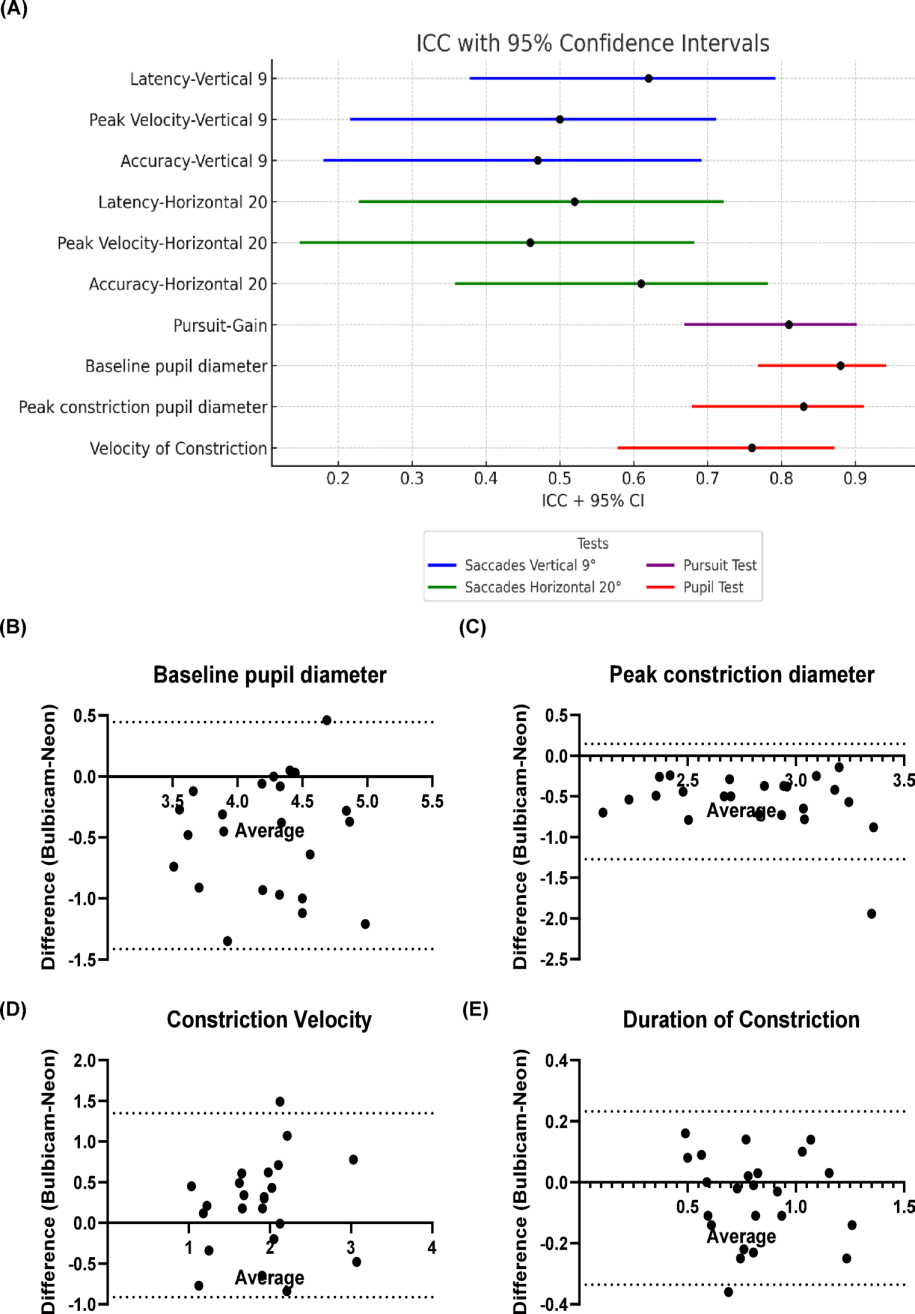
Intra-Class Correlation (ICC) Analysis and Bland-Altman Plots: (**A**) ICC values with 95% confidence intervals for saccades, pursuit and pupil tests. Colours represent different test groups: vertical 9°(degree) saccadic task (blue), horizontal 20°(degree) saccadic task (green), pursuit test (purple), and pupil tests (red). (**B-E**) Bland-Altman plots for the pupil test metrics for pupil diameter (baseline and peak constriction), constriction velocity (mm/s) and duration of constriction. Average represents the bias between two measurements, while dashed lines indicate the 95% limits of agreement (LoA).

The pursuit test demonstrated high reproducibility, with an ICC value of 0.81 (95% CI:0.67–0.90) (Fig. 4 A). For the vertical 9 degree saccades test, the ICC values for latency, peak velocity, and accuracy were 0.62 (95% CI:0.38–0.79), 0.50 (95% CI:0.22–0.71), and 0.49 (95% CI: 0.18–0.69), respectively. Similarly, for the horizontal 20 degree saccades test, the ICC values for these metrics were 0.52 (95% CI:0.23–0.72), 0.46(95% CI:0.15–0.68), and 0.61(95% CI:0.36–0.78), respectively (Fig. 4 A).

In the pupil test, high reproducibility was observed for the baseline pupil diameter (ICC: 0.88, 95% CI:0.77–0.94), for the peak constriction diameter (ICC: 0.83, 95% CI:0.68–0.91), as well as for the constriction velocity (ICC: 0.76, 95% CI:0.58–0.87) (Fig. 4 A).

These results indicate varying levels of reproducibility across the tested parameters, with particularly strong consistency in the pursuit and pupil test metrics.

### Paired assessment between devices

Bland-Altman plots illustrate the agreement between BulbiCAM and PupilLabs Neon glasses measurements across various pupil test parameters. Paired assessment between devices showed close agreement for key pupillometer metrics: baseline diameter (bias: −0.48 ± 0.47 mm LoA:−1.4 to 0.45), peak constriction diameter (bias: −0.56 ± 0.36 mm LoA: −1.3 to 0.15), constriction velocity (bias: 0.22 ± 0.58 mm/s LoA: −0.9 to 1.3), and duration of constriction (bias: −0.052 ± 0.15 s LoA: −0.34 to 0.23). Most data points fell within the limits of agreement (LoA), demonstrating agreement between systems (Fig. 4B, 4 C, 4D, 4E). These results suggest good agreement between the two systems, with minor, consistent biases across static and dynamic pupil measurement parameters.

## Discussion

This study evaluated the technical feasibility of the BulbiCAM device in healthy participants, providing baseline metrics before clinical validation, focusing on the reproducibility of pursuit, saccade, and pupillometry tests. In addition, a paired assessment between BulbiCAM and the PupilLabs Neon eye-tracking glasses examined the agreement between devices. Overall, participant feedback indicated a high level of acceptability, with favourable ratings for comfort, examination length, brightness, and minimal reports of double vision or eye strain. Smooth pursuit and pupillometry tests demonstrated strong reproducibility, while saccade tests showed moderate to poor results. These findings establish BulbiCAM’s technical performance and participant acceptability in a healthy cohort, supporting the potential for clinical applicability following patient-based validation.

The pursuit test showed good reproducibility at 10-degree, 0.5 Hz test frequency. The reproducibility of the BulbiCAM was assessed using the gain value of the pursuit test at a stimulus frequency of 0.5 Hz, as pursuit gain is known to decrease at frequencies above 0.5 Hz^18^. Although previous studies have shown that gain values at 0.25 Hz are higher than those at 0.5 Hz in children, with performance declining as target velocity increases, our findings confirm the high reproducibility of the pursuit test at 0.5 Hz. This suggests that 0.5 Hz is an ideal parameter for reproducibility assessments^19,20^.

Inter-visit reproducibility for pupillary light reaction tests showed good reproducibility for baseline-peak constriction diameters and constriction velocity parameters.

In contrast, the latency, peak velocity, and accuracy parameters of the vertical 9 and horizontal 20 saccade tests showed only moderate to poor reproducibility. These findings are in line with a recent technical report of BulbiCAM’s utility in a Parkinson’s disease cohort^21^. This discrepancy in reproducibility between the saccadic and pupillometry tests can be attributed to fundamental differences in the physiological mechanisms underlying these tasks. Pupillary light reflexes are primarily mediated by the autonomic nervous system, which is relatively stable and less influenced by higher-order cognitive processes. In contrast, saccadic eye movements are complex, involving the integration of sensory, cognitive, and motor systems. Factors such as attention, learning effects, and decision-making are known to influence saccadic performance, potentially introducing variability between visits^22–24^. Beyond participant factors, device characteristics may also limit reliability of high-frequency oculomotor metrics. At 400 Hz the 2.5 millisecond (ms) frame period sets a latency quantisation floor (± 1.25 ms), and we observed intermittent doubled inter-frame intervals (~ 4.7–4.9 ms) that add low-amplitude jitter. Together with small calibration error/drift and the camera-side spatial scale (~ 2.1 px/°), these factors likely contribute to the moderate ICC observed. Therefore, the moderate to poor reproducibility observed in our study may reflect individual differences in attention and engagement across visits as well as the technical limitations of the device.

Notably, the pursuit tasks exhibited less variability, despite also being influenced by attention and engagement. This difference may be attributed to the pursuit system’s reliance on continuous, reflexive tracking mechanisms, which are less susceptible to transient fluctuations in attention, cognitive engagement, or physiological states compared to saccadic movements. Additionally, the study design, which required two separate visits, could have introduced variability due to learning effects or differences in participant familiarity with the task. The use of a VR-based testing system, such as the BulbiCAM, might also play a role. While effective for certain measures, it may not be optimized for capturing the fine-grained dynamics of saccadic eye movements with high reproducibility. Moreover, headset fit, ambient illumination, and calibration drift represent potential limiting factors contributing to lower reproducibility in VR-based eye tracking. Optimal headset fit is essential for accurate gaze detection and measurement precision, as poor alignment or shifting of the device during testing can degrade data quality. Calibration drift, which refers to gradual changes in the eye tracker’s calibration over time, can result in loss of data accuracy if not detected and corrected regularly^25^. Future studies could explore modifications to the testing protocol or alternative setups to improve the reliability of saccade measurements using VR-based systems.

This study demonstrates agreement between devices in pupillometry test variables, indicating reliable measurements across the observed parameters. BulbiCAM consistently measured lower pupil diameters for both baseline and peak constriction states, compared to PupilLabs Neon glasses. These differences can be attributed to variations in participants’ head positioning during the assessments. During Neon glasses testing, participants’ heads were positioned slightly back, leading to variable brightness levels. In contrast, BulbiCAM assessments ensured more consistent head positioning, resulting in brighter light exposure and consequently smaller pupil diameters, particularly at the peak constriction phase. Moreover, repeated exposure to bright light stimuli during the trials, is likely to influence any measure of ‘baseline’ diameter. This effect, known as the post-illumination pupil response, reflects the delay in returning to baseline dilation after exposure to a stimulus, which can take several seconds^26^. Consequently, differences in stimulus illumination levels contributed to the variation in baseline pupil diameter in this study.

Discrepancies in baseline measurements between BulbiCAM and PupilLabs Neon glasses may also be attributed to variation in head positioning and light illuminance^27^.

Despite these technical differences, Bland-Altman analysis showed agreement between BulbiCAM and Neon in baseline and peak constriction pupil diameter, with biases of approximately 0.5 mm with few observations near or outside the limits of agreement (LoA:−1.4 to 0.45 and LoA: −1.3 to 0.15 respectively). Thus, while careful interpretation is warranted, these differences in pupil diameter are consistent with those reported in various clinical cohorts, ranging from healthy participants to comatose, critically ill cardiac patients^28,29^. Taken together, our findings and prior evidence suggest that, despite variability from head positioning and stimulus illuminance, the agreement between BulbiCAM and PupilLabs Neon glasses for pupil diameter measurements is robust in healthy participants and may support future application in clinical settings.

The BulbiCAM system also measures a higher velocity of pupil constriction compared to Neon glasses. Pupil velocity is determined by identifying a consistent constriction or dilation phase and calculating the change in pupil diameter or area divided by the phase duration^13^. Velocity, defined as the change in pupil diameter per second for this test, is greater in BulbiCAM because the diameter change (absolute value of baseline-peak constriction difference) is larger, and the time to reach peak constriction is faster. Neon glasses recorded a longer time to constriction, likely due to lower brightness levels during the assessment, which may have caused a slower and more gradual pupil constriction compared to the sharper response observed with BulbiCAM. Therefore, while BulbiCAM shows higher velocity measurements, considering the diameter change and time to constriction variables used to calculate velocity, BulbiCAM’s velocity measurement is still acceptable and reliable for pupil testing.

Although BulbiCAM showed strong reproducibility for pupillometry and pursuit metrics as well as high acceptability in healthy individuals, true clinical feasibility and utility can only be determined by extending validation to patient populations with neuro-ophthalmic disorders with future studies. These should include individuals with neuro-ophthalmic disorders such as optic neuritis, papilledema, and cranial nerve palsies, as well as other conditions affecting pupil and pursuit metrics. Sample sizes should be adequately powered to assess clinical validity, with the design and sample size of the present study serving as a useful reference. Primary outcome measures should include the reproducibility and reliability of saccade, pupillometry and pursuit metrics in patient cohorts, alongside sensitivity and specificity compared with established reference-standard diagnostic methods.

We additionally acknowledge some limitations of this study. Firstly, reliability was assessed solely for the paired comparison of the pupil test between BulbiCAM and PupilLabs Neon glasses. No evaluations involving other devices or tests were conducted, as replicating the exact stimulus was not feasible with a traditional infrared eye tracker. Second, the recent updates to the device software, require further testing to assess their impact on reproducibility and reliability. Moreover, some internal device limitations have been reported previously, limiting saccade test reproducibility.

## Conclusion

This study demonstrates the technical reproducibility, and reliability of BulbiCAM in healthy participants. These findings provide foundational metrics, but further studies are needed to validate BulbiCAM’s clinical feasibility and utility in patient populations. Participant feedback on the BulbiCAM examination was highly positive. Inter-visit reproducibility was high for pursuit and pupil tests, supporting the device’s consistency. While saccades showed poor to moderate reproducibility, this highlights areas for potential refinement and investigating further. The paired assessment between devices further validated the accuracy of key pupillometric parameter. These results underscore the potential of BulbiCAM as a reliable and patient-friendly device for oculomotor and pupillometric evaluations in both clinical and research settings.

The findings of this study provide a foundation for future research into the application of VR-based eye-tracking systems across various disorders and contexts. In the future, we foresee portable VR based headset eye tracking devices playing a significant role in meeting the increasing need for imaging across diverse settings, confirming the point-of-care approach.

## Supplementary Information

Below is the link to the electronic supplementary material.

Supplementary Material 1

## Data Availability

Researchers interested in accessing the data are encouraged to contact the corresponding author to discuss individual access arrangements.

## References

[1] Oyama, A. et al. Novel method for rapid assessment of cognitive impairment using High-Performance Eye-Tracking technology. *Sci. Rep.* **9**, 12932 (2019). 10.1038/s41598-019-49275-x PMC6736938 31506486

[2] Kim, M. et al. Development of an eye-tracking system based on a deep learning model to assess executive function in patients with mental illnesses. *Sci. Rep.* **14**, 18186 (2024). 10.1038/s41598-024-68586-2 PMC11303748 39107349

[3] Leigh, R. J. & Kennard, C. Using saccades as a research tool in the clinical neurosciences. *Brain* **127** (3), 460–477 (2004). 14607787 10.1093/brain/awh035

[4] Xu, Y., Zhang, C., Pan, B., Yuan, Q. & Zhang, X. A portable and efficient dementia screening tool using eye tracking machine learning and virtual reality. *NPJ Digit. Med.* **7, **219 (2024). 10.1038/s41746-024-01206-5 PMC11341897 39174736

[5] Moreno-Arjonilla J, López-Ruiz A, Jiménez-Pérez JR, Callejas-Aguilera JE, Jurado JM. Eye-tracking on virtual reality: a survey. *Virtual Reality*.**28**. 10.1007/s10055-023-00903-y (2024).

[6] Hirota, M. et al. Analysis of smooth pursuit eye movements in a clinical context by tracking the target and eyes. *Sci. Rep.* **12**, 8501 (2022). 35589979 10.1038/s41598-022-12630-6 PMC9120200

[7] Soler-Dominguez, J., Cavalcanti, J., Contero, M. & Alcañiz Raya, M. The power of sight: Using eye tracking to assess learning experience (LX) in virtual reality environments. 11th International Technology, Education and Development Conference (INTED2017) Proceedings (2017).

[8] Barkevich, K., Bailey, R. & Diaz, G. J. Using deep learning to increase Eye-Tracking Robustness, Accuracy, and precision in virtual reality. *Proc. ACM Comput. Graph Interact. Tech.* **7** (2), 10.1145/3654705 (2024). PMID: 39119010; PMCID: PMC11308822. 10.1145/3654705 PMC11308822 39119010

[9] Bulbitech user manual. ; (2023). Available at: https://bulbitech.com/

[10] Coito, A. et al. Test-retest reliability of gaze precision of a novel virtual reality-based medical device. *Front. Virtual Real.* **6**, 1502679. 10.3389/frvir.2025.1502679 (2025).

[11] Sarker, P. et al. Test–retest reliability of virtual reality devices in quantifying for relative afferent pupillary defect. *Transl Vis. Sci. Technol.* **12** (6), 2. 10.1167/tvst.12.6.2 (2023). 37279393 10.1167/tvst.12.6.2 PMC10249680

[12] Pupil Labs. Pupil Labs Neon. ; (2024). Available at: https://pupil-labs.com/products/neon

[13] Holmqvist K, Nyström M, Andersson R, Dewhurst R, Jarodzka H, van de Weijer J. Eye Tracking: A comprehensive guide to methods and measures. Oxford: OUP Oxford; 2011.

[14] Blekher, T. et al. Test-retest reliability of saccadic measures in subjects at risk for huntington disease. *Invest. Ophthalmol. Vis. Sci.* **50** (12), 5707–5711 (2009). 19553607 10.1167/iovs.09-3538

[15] Weir, J. P. Quantifying test-retest reliability using the intraclass correlation coefficient and the SEM. *J. Strength. Cond Res.* **19** (1), 231–240 (2005). 15705040 10.1519/15184.1

[16] Nij Bijvank, J. A. et al. A standardized protocol for quantification of saccadic eye movements: demons. *PLoS One*. **13** (7), e0200695 (2018). 30011322 10.1371/journal.pone.0200695 PMC6047815

[17] Bland, J. M. & Altman, D. G. Statistical methods for assessing agreement between two methods of clinical measurement. Lancet 327, 307–310 (1986). 2868172

[18] Martins, A. J., Kowler, E. & Palmer, C. Smooth pursuit of small-amplitude sinusoidal motion. *J. Opt. Soc. Am. A*. **2**, 234–242 (1985). 3973755 10.1364/josaa.2.000234

[19] Salman, M. S., Sharpe, J. A., Lillakas, L., Dennis, M. & Steinbach, M. J. Smooth pursuit eye movements in children. *Exp. Brain Res.* **169**, 139–143 (2006). 16362363 10.1007/s00221-005-0292-7

[20] Schröder, R., Keidel, K., Trautner, P., Radbruch, A. & Ettinger, U. Neural mechanisms of background and velocity effects in smooth pursuit eye movements. *Hum. Brain Mapp.* **44** (3), 1002–1018 (2023). 36331125 10.1002/hbm.26127 PMC9875926

[21] Dalbro, S. E. J. et al. Repeatability, reliability, and stability of eye movement measurements in parkinson’s disease, cerebellar ataxia, and healthy adults. *Front. Neurol.* **16, **1556314 10.3389/fneur.2025.1556314 (2025). 10.3389/fneur.2025.1556314 PMC12068062 40356635

[22] Gersch, T. M., Kowler, E., Schnitzer, B. S. & Dosher, B. A. Attention during sequences of saccades along marked and memorized paths. *Vis. Res.* **49** (10), 1256–1266 (2009). 18226827 10.1016/j.visres.2007.10.030 PMC3516297

[23] Hutton, S. B. Cognitive control of saccadic eye movements. *Brain Cogn.* **68** (3), 327–340 (2008). 19028265 10.1016/j.bandc.2008.08.021

[24] Pierce JE, Clementz BA, McDowell JE. Saccades: fundamentals and neural mechanisms. In: Klein C, Ettinger U (eds). Eye Movement Research. Studies in Neuroscience, Psychology and Behavioral Economics. Cham: Springer, 11–71 10.1007/978-3-030-20085-5_2(2019).

[25] Al Madi, N. On the validity and benefit of manual and automated drift correction in reading tasks. *J. Eye Mov. Res.* **18** (3), 17. 10.3390/jemr18030017 (2025). PMID: 40417429; PMCID: PMC12101172. 40417429 10.3390/jemr18030017 PMC12101172

[26] Mathôt, S. Pupillometry: Psychology, Physiology, and function. *J. Cogn.* **1** (1), 16 (2018). 31517190 10.5334/joc.18 PMC6634360

[27] Wickremasinghe, S. S., Smith, G. T. & Stevens, J. D. Comparison of dynamic digital pupillometry and static measurements of pupil size in determining scotopic pupil size before refractive surgery. *J. Cataract Refract. Surg.* **31** (6), 1171–1176 (2005). 16039493 10.1016/j.jcrs.2004.10.049

[28] Schmitz, S., Krummenauer, F., Henn, S. & Dick, H. B. Comparison of three different technologies for pupil diameter measurement. *Graefes Arch. Clin. Exp. Ophthalmol.* **241** (6), 472–477 (2003). 12739174 10.1007/s00417-003-0669-x

[29] Nyholm, B. et al. Superior reproducibility and repeatability in automated quantitative pupillometry compared to standard manual assessment, and quantitative pupillary response parameters present high reliability in critically ill cardiac patients. *PLoS One*. **17** (7), e0272303. 10.1371/journal.pone.0272303 (2022). PMID: 35901103; PMCID: PMC9333219. 35901103 10.1371/journal.pone.0272303 PMC9333219

