# Supplementary material for: Technical validation of a virtual reality-based eye tracker for neuro-ophthalmic assessment: a reliability and reproducibility study
Source: Sci Rep. 2026 Jan 5;16:1134. doi: 10.1038/s41598-025-30773-0 (PMC12789551; doi:10.1038/s41598-025-30773-0)
Supplement: Supplementary file 1 — Supplementary Material 1 [file 41598_2025_30773_MOESM1_ESM.pdf]

Supplementary Information: Technical Validation Metrics for Eye-Tracking System BulbiCAM

This supplementary document provides the comprehensive technical validation metrics required for reproducibility and comparability with standard eye-tracking validation studies. The following sections detail (a) spatial accuracy and precision, (b) temporal resolution, (c) calibration accuracy, and (d) AI-based pupil detection validation.

(a) Spatial Accuracy and Precision Measurements

Display and Camera Geometry:

- Viewing distance: 147.5 mm
- Display resolution and size: 2560×1440 pixels over 120.96×68.04 mm physical screen
- Display scale: 2.57 mm/° → 54.49 pixels/° (display)
- Target stimulus size: 30-pixel white dot ≈ 0.55° visual angle (Goldmann III ≈ 0.43°)
- Eye-tracker camera scale (eye-plane): ≈0.10 mm/pixel with ~500 px region of interest (ROI) spanning ~50 mm palpebral width
- Ocular rotation radius: ~12 mm → small angle mapping 0.48°/pixel (~2.1 px/°; range 0.458° to 0.498°/pixel for rotation radii between 11.5–12.5 mm)

Accuracy and Precision Calculation:

- Accuracy and precision in degrees should be computed from calibration and fixation residuals from the eye tracker using 0.48°/px.
- Precision is quantified as the standard deviation (SD) or 68% confidence interval of fixation noise, central gaze (0–5°) and peripheral gaze (>5°) after conversion.

(b) Temporal Resolution Validation

Actual Achieved Sampling vs Nominal Rates:

- The system nominally samples at 400 Hz (frame period 2.50 ms; quantisation error ±1.25 ms).
- Measured from three representative timestamp series:
    - Median inter-frame interval: ≈ 2.38 ms (achieved median ~420 Hz).
    - Interquartile range (IQR) of inter-frame intervals from 2.27 ms to 2.50 ms, indicating tight timing consistency.

Dropped Frames and Data Loss:

- Intermittent doubled intervals (~4.7–4.9 ms) occur as short bursts (OS/USB scheduling), not sustained loss, with no evidence of persistent dropped frames or buffer overrun in these samples.
- Temporal resolution validation showing actual achieved sampling rates versus nominal rates, including analysis of dropped frames and data loss percentages; synchronization between right and left eye data is perfectly compensated due to single-camera setup.
- Stimulus-to-eye timing accuracy is supported by calibration tool step 2 based on 100 and 1000 samples, crucial for saccadic response time (SRT) analysis whereby SRTs are compensated by average values.

(c) Calibration Accuracy Metrics; RMS errors, maximum error, accuracy degradation over time

Per-target residuals (in pixels) were extracted from the calibration and verification logs (including any mid- or post-run checks).
All residuals were converted to degrees of visual angle using the camera-side conversion factor of **0.48°/px**.

From these converted values, the following metrics were calculated:

- **RMS error (°)** and **maximum error (°)**.
- **Drift (°)**, defined as the change in RMS error between pre- and post-calibration (or late-run fixation check).

The conversion factor is calculated; RMS, maximum error, and drift values were computed directly from calibration logs and reported separately for **central** and **peripheral** targets where applicable.

Note on System Comparison:

- Glint-only tracking systems (e.g., BulbiCAM) outperform combined pupil+glint methods in accuracy, due to lower noise in glint position data compared to pupil/ellipse data based on AI pupil detection, especially in large gaze.

(d) Cross-Validation of AI Pupil Detection Algorithms; including sensitivity, specifity, failure mode analysis

Methodology:

- Cross-validation was performed on stratified random samples across lighting, gaze position, blinks, and motion.

- Manual adjudication was performed to label pupils as **valid pupil detected** or **invalid/occluded**. These labels were compared with the device's automated detection flag to calculate **sensitivity** and **specificity** of the pupil detection algorithm.
- From a 100-frame subset, pupil centers are manually annotated to compute **centre-localization errors (°)** via 0.48°/px scaling.

Failure modes identified include:

- Blinks and partial blinks
- Dense eyelashes or mascara
- Overlap of glint with edge
- Low infrared (IR) contrast, especially in dark irides
- Extreme gaze angles or motion blur

Since pupil detection is not used for eye-tracking measurements, any noise in pupil data affects only the **pupillometry test** and does not influence other analyses.
